# The role of changing temperature in microbial metabolic processes during permafrost thaw

**DOI:** 10.1371/journal.pone.0232169

**Published:** 2020-04-30

**Authors:** Komi S. Messan, Robert M. Jones, Stacey J. Doherty, Karen Foley, Thomas A. Douglas, Robyn A. Barbato

**Affiliations:** 1 US Army Engineer Research and Development Center, Cold Regions Research and Engineering Laboratory, Hanover, New Hampshire, United States of America; 2 US Army Engineer Research and Development Center, Cold Regions Research and Engineering Laboratory, Fairbanks, Alaska, United States of America; Central South University, CHINA

## Abstract

Approximately one fourth of the Earth’s Northern Hemisphere is underlain by permafrost, earth materials (soil, organic matter, or bedrock), that has been continuously frozen for at least two consecutive years. Numerous studies point to evidence of accelerated climate warming in the Arctic and sub-Arctic where permafrost is located. Changes to permafrost biochemical processes may critically impact ecosystem processes at the landscape scale. Here, we sought to understand how the permafrost metabolome responds to thaw and how this response differs based on location (i.e. chronosequence of permafrost formation constituting diverse permafrost types). We analyzed metabolites from microbial cells originating from Alaskan permafrost. Overall, permafrost thaw induced a shift in microbial metabolic processes. Of note were the dissimilarities in biochemical structure between frozen and thawed samples. The thawed permafrost metabolomes from different locations were highly similar. In the intact permafrost, several metabolites with antagonist properties were identified, illustrating the competitive survival strategy required to survive a frozen state. Interestingly, the intensity of these antagonistic metabolites decreased with warmer temperature, indicating a shift in ecological strategies in thawed permafrost. These findings illustrate the impact of change in temperature and spatial variability as permafrost undergoes thaw, knowledge that will become crucial for predicting permafrost biogeochemical dynamics as the Arctic and Antarctic landscapes continue to warm.

## Introduction

High latitude regions (i.e. Antarctica, alpine regions, Arctic regions of Siberia, Greenland, Alaska, Canada, etc.) are warming at unprecedented rates as global temperatures continue to rise, resulting in vulnerability to permafrost (i.e. soil, organic matter, or bedrock that has been frozen for at least two consecutive years) through thaw and degradation [1–7, 8]. The outcomes of climate warming are diverse, encompassing both physical and biological changes across the region. Physical changes include sea ice reduction, increasing or decreasing formation of lakes, ponds, and wetlands, and even severe damage to buildings and infrastructure in the Arctic [9–12]. Biological changes include a rise in atmospheric carbon dioxide and methane due to increased biological activity, change in vegetation type, and intensification of toxic cyanobacteria blooms [13–17]. Permafrost underlies one fourth of the Northern Hemisphere [18] and the acceleration of its warming has diverse effects on the ecosystem. Of primary concern is the immediate change to the productivity of microorganisms embedded within permafrost and the uncertainty of positive feedbacks to global warming. Though it is well known microorganisms can survive under permafrost conditions over a geologically significant time [19–23] and that they are adept to respond to permafrost thaw [24–27], questions remain regarding the similarities in the microbial functional response at different permafrost locations.

Early studies revealed that a variety of viable microorganisms are present in permafrost from several Arctic and Antarctic sites [21, 22, 28–30;8]. For instance, a wide diversity of bacteria including aerobic and anaerobic heterotrophs, methanogens, iron reducers, sulfate reducers, nitrifying and nitrogen fixing bacteria have been successfully isolated from permafrost soils [20, 28, 31]. Moreover, both microbial community structure and functional response have been observed to shift following thaw at single sample sites [24–26, 32, 33]. Specifically, community structure diverged with increased temperature [24, 33] or thaw stage [25, 26]. However, in a study of permafrost collected in interior Alaska, microbial functional potential converged with thaw [24], suggesting divergent community responses to thaw may have similar functional outcomes. Furthermore, microbial activity and the production and emission of greenhouse gases such as carbon dioxide (CO_2_), methane (CH_4_), and nitrogen oxide have been shown to increase following permafrost thaw (N_2_O [32], reviewed in Ernakovich et al. [33], Graham et al. [34], Stackhouse et al. [35], [36, 37, 38]. Although permafrost often acts as a source of carbon emissions post-thaw, active atmospheric methane-utilizing bacteria (methanotrophs) use CH_4_, resulting in declines in emission rates under extended periods of warm temperature [24, 35, 39].

While studies using marker gene and metagenomics analysis provide valuable insight into the shift of permafrost microbial communities and potential functions driven by climate warming, other ‘omics technologies (e.g. metaproteomics, metabolomics, etc.) offer unique insight into the active processes conducted by microorganisms. Advances in measurement techniques and computer performance has resulted in considerable progress in metabolic studies (i.e. better data processing capabilities) of environmental phenomena [40, 41]. Investigating microbial metabolic profiles reveals active processes across the community as these metabolites are intermediates and important products of metabolism. For instance, microbial metabolites have important ecosystem functions in soil by controlling plant disease [42–44], stimulation of other processes such as denitrification [45–47], and antibiotic production for competition or mediating communication [48–50]. This method has been specifically used in environmental studies to determine the effects of drought stress over *Arabidopsis thaliana* [51], to assess toxicodynamics and threshold effect levels of environmental pollutants in aquatic organisms [52], and to identify the nutritional differences between traditional and genetically modified crops [53].

Metabolites also regulate microbial community interactions by promoting or suppressing growth of other individuals. These mutualistic and antagonistic relationships likely play a significant role in community assembly. As thaw accelerates due to the changing climate, shifts in metabolite production will directly affect ecosystem function and soil health [34, 54]. However, this technology has yet to be used to detect metabolic changes occurring within thawing permafrost thus omitting important microorganisms’ responses in permafrost induced by environmental stressors such as increased temperature.

The purpose of this study was to examine metabolic changes in the in situ microbial communities from permafrost collected at three different physical locations (35 m, 60 m, and 83 m) in the Cold Regions Research and Engineering Laboratory’s Permafrost Tunnel located in Fox, Alaska. The Tunnel provides access to permafrost with different deposition histories and ages which represents a variable landscape. The permafrost core samples were incubated aerobically under controlled conditions at -3°C and 6°C to account for pre- and post-thaw conditions that represent current permafrost and active layer temperatures in interior Alaska, respectively. Metabolic profiling was conducted on microbial cells extracted from the incubated samples to illustrate spatial variability and to identify biogeochemical dynamics associated with thaw. To the best of our knowledge, this is the first study to illustrate the functional response of the permafrost microbiome during thaw using an untargeted metabolomics analysis. Spatial distance constitutes an important factor in the prediction of bacterial and archaeal beta diversity in seasonally thawed (“active layer”) and permafrost soils [55, 56]. To understand the influence of spatial heterogeneity on functional outcomes of permafrost thaw, we assessed permafrost community response to thawing temperature and examined whether this response may differ over heterogenous soil or landscape characteristics using univariate and multivariate statistical analyses.

## Materials and methods

### Experimental design

All permafrost samples were collected from the U.S. Army Cold Regions Research and Engineering Laboratory’s Permafrost Tunnel located in Fox, Alaska (64.9528^o^ N, -147.6178^o^ W; hereafter referred to as “the Tunnel”) in July 2017. The Tunnel includes two parallel excavations, each roughly 100 m long, 8 m high, and 4 to 5 m wide. The main (North) tunnel was excavated in the mid-1960s [57] but a newer parallel excavation (South) was initiated from 2011 to 2018. The Tunnel presents a stable air temperature (-4.1 ± 1.4°C) and relative humidity of 91 ± 3% [58]. The sample we collected represent permafrost that has not thawed since it was deposited. Permafrost accessible in the Tunnel is syngenetic ice cemented loess that represents the high carbon and ice content “yedoma” type permafrost present across Interior Alaska and parts of Siberia [59]. This type of permafrost is vulnerable to thaw because of its high ice content and it contains vast stores of carbon rich soils.

At each permafrost sample location (35 m, 60 m, and 83 m from the North Tunnel portal), triplicate cores were collected by drilling 1 m horizontally into the permafrost tunnel wall using a SIPRE corer. This allowed us to capture different representations of loess deposition representing various ages with increasing distance in the Tunnel (see samples collected from the tunnel at 20 m, 54 m, 81 m with their respective ages, 19000 y.b.p, 27000 y.b.p., and 33000 y.b.p. in Mackelprang et al. [23]). Since syngenetic permafrost aggrades upward over time, the different aged soils in our sample suite represent different climatic (and thus soil biogeochemical characteristics) over a 14000 year period [60]. Cores were shipped frozen to the Cold Regions Research and Engineering Laboratory in Hanover, NH and were stored frozen.

At the start of the experiment, the cores were prepared to remove exogenous cells and molecules by removing the outer portion of the core and aseptically drilling new, untouched sub-samples following protocols of Barbato et al. [61] in a -10°C cold room. To achieve a 10 g sub-sample, clean sub-samples of permafrost from each core location were consolidated. In total, six replicates of permafrost were used from each location to yield a total of 18 samples for incubation (i.e. six 10 g samples at each location of 35 m, 60 m, and 83 m). Half of the samples were incubated at -3°C to represent *in situ* permafrost temperatures in the Tunnel [58]. The other half of the samples were incubated at 6°C to represent active layer temperatures in interior Alaska at a nearby site (Barbato, unpublished data) to achieve a total of two incubation temperatures. All samples were incubated aerobically in the dark in two separate Yamato Scientific Low Temperature Incubators (IN601, Tokyo, Japan) for 120 hours. The sample at 6°C thawed into a wet silt that resembled organic rich muddy soil. After the incubation period, each 10 g sample was individually suspended in 95 ml of 1°C filter-sterilized (0.22 μm) sodium pyrophosphate buffer and mixed on a horizontal shaker table (Eberbach E5850.C.15, USA) at 1°C for 1 hour to keep the samples in a constant cold temperature without the potential of damaging the cells at a freezing temperature [62]. The sodium pyrophosphate buffer was utilized to dissociate soil particles and liberate the microbial cells into solution. Larger particles were allowed to settle undisturbed for 10 minutes and then equal volumes of the samples were divided into two centrifuge tubes with a pipette and centrifuged at 8000xg for 5 minutes at 4°C. The supernatant was decanted and the pellet was re-suspended in 10 ml of 1°C filter sterilized (0.22 μm) phosphate buffered saline before it was recombined with the other volume separated previously. The combined material was then centrifuged for 5 minutes at 8000xg at 4°C then the supernatant was decanted and the pellet re-suspended again with 10 ml of cold filter sterilized phosphate buffered saline. The pellet was centrifuged one final time under the same conditions mentioned previously and the supernatant was pipetted off before the remaining cell pellet was frozen and stored at -20°C until metabolite analysis was performed.

### Metabolite profiling by UPLC-Q-TOF/MS

Metabolome profiling was conducted by Creative Proteomics (New York, USA). Ten ml of ultrapure (18.2 M-ohm) water was added to the frozen cell pellets. The mixture was placed in an ice-water bath ultrasound apparatus for 2 minutes (break for 15 seconds, stop for 10 seconds), and then centrifuged at 13000 rpm at 4°C for 15 minutes. The supernatant was analyzed by an Ultra Performance Liquid Chromatography- Quadrupole / Time-Of-Flight / Mass Spectrometer (UPLC-Q/TOF/MS; Xevo G2 Waters, USA).

Sample separation was performed by UPLC (Waters, USA) and screened with Electron Spray Ionization (ESI)-MS (untargeted MS/MS mode). A 5 μl aliquot of rat plasma sample was injected onto an ACQUITY UPLC HSS C18 column (2.1 mm x 100 mm, 1.7 μm, Waters). The mobile phase was composed of solvent A (0.1% formic acid in water) and solvent B (0.1% formic acid acetonitrile) with a gradient elution (0–5 minutes, 95–75% A; 5–22 minutes, 70–1% A; 22–25 minutes, 1% A). The flow rate of the mobile phase was 0.5 ml/minute and the sample manager temperature was set at 4°C.

Mass spectrometry was performed on a Q-TOF Mass Spectrometer using a Dual Agilent Jet Stream (AJS) ESI source. The scanning mass-to-charge (m/z) range is from 50 to 1500 with a scan frequency of 15 seconds. The capillary voltage was set to 3000 V and 2000 V (positive and negative mode, respectively) and the fragmentor was set to 175 V. The pressure of the nebulizer was set at 35 psi, the gas temperature was set to 450°C, and the continuous gas flow was 0.3 ml/minute. The instrument was set to Resolution mode.

**Fig 1:**
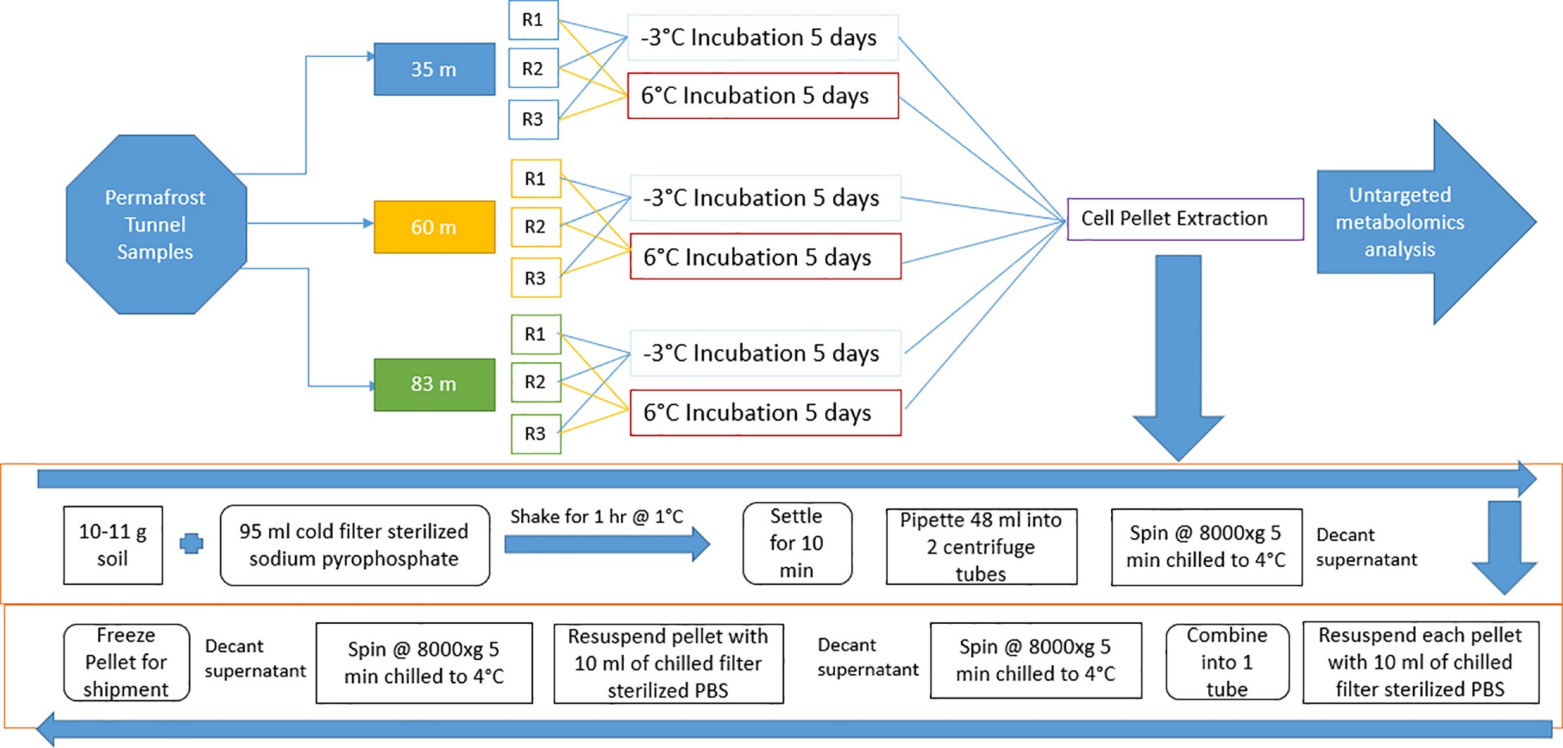
Schematic diagram of the procedure used for the experimental set-up and metabolome extraction. PBS denotes Phosphate Buffered Saline.

LC-MS data were obtained using Masslynx (Waters, USA). Makerlynx software (version 4.1) was then used to extract ion pairs, align matching peaks, and determine peak intensity for the corrected result. More specifically, chromatographic peaks were detected between a retention time of 0 and 30 minutes. Data composed of spectrometric attributes such as retention time and mass-to-charge were generated and the corresponding relative intensity was also provided for each sample. A signal to noise ratio of five was used as a threshold value to reduce noise in the data. In order to prevent artifacts within the data and have consistent intensities between samples, the intensity values were normalized to the Total Ion Current (TIC) where the summation of all attributes (i.e. retention time and mass-to-charge) yielded 10000, for all samples (see similar procedure in Wallenstein et al. [63]. All raw data are publicly available in the MetaboLights database [64] and a summary of our experimental design along with the metabolic profiling is illustrated in the schematic diagram in Fig 1.

### Data analysis

The raw data were acquired based on the m/z value and the retention time of the ion signals. Ions from both ESI- and ESI+ were extracted for multivariate analysis using R software [65]. A cutoff p-value < 0.05 was used to establish statistical significant differences for all comparisons. Methodologies described in Grace and Hudson [66] were utilized to preprocess the data for optimum results in both ESI+ and ESI- modes. All data preprocessing and statistical analysis were performed using signal intensities from ESI+ and ESI-. The values were log transformed to remove heteroscedasticity and correct for skewness in the data distribution Moreover, the data were mean-centered and pareto-scaled given that scaling (or normalizing) the data makes the samples more comparable to each other [66, 67]. Following data preprocessing, global metabolism variations were investigated by first using Principal Component Analysis (PCA) to study observations acquired in ion modes. PCA as an unsupervised method was employed here due to its low noise sensitivity and increased efficiency of detecting features [68]. Noting that metabolites with high PCA loadings in magnitude (i.e. positive or negative) are important for discriminating groups in the PCA scores [66], any variable in the top or lower 10% of the PCA loadings were considered to be the significant metabolites driving the group discrimination.

Since PCA results can be masked by high within group variation that renders them unsatisfactory [66, 69], a Partial Least Square Discriminant Analysis (PLS-DA) was also performed to sharpen the separation between groups and identify potential biomarkers. The biomarkers were filtered and confirmed using the Variable Importance in the Projection (VIP) value (VIP>1.5). The VIP score is often used for variable selection as this method selects the predictor variables that contribute the most to the underlying variation in the response variable [70, 71]. The pre and post filtered data are referred to as the “unfiltered dataset” and “filtered dataset”, respectively. Chemical structures of metabolites in the filtered dataset were identified according to online databases that include the Human Metabolome Database (www.hmdb.ca), Meltin (www.meltin.scripps.edu), and the Mass Bank (www.massbank.jp) using the data accurate masses and MS/MS fragments. When necessary, further confirmation was acquired through comparison with authentic standards with focus on retention times and MS/MS fragmentation patterns. Noting that features in the unfiltered dataset could also explain within group variation, the data preprocessing, multivariate analysis, and significant metabolites identification methodology used on the unfiltered dataset was also employed on the filtered dataset.

Additional statistical analysis was conducted to compare intensity differences of known metabolites within the sample groups in the filtered dataset. A paired samples Wilcoxon t-test was conducted to measure statistically significant differences between the significant metabolites present at -3°C and 6°C. Moreover, a one-way Analysis of Variance (ANOVA) was used to compare differences in metabolites within the sample locations (i.e. 35 m, 60 m, and 83 m) for both the -3°C and 6°C incubations. For the ANOVA results, adherence to normality and homogeneity of variance were verified using Shapiro-Wilk’s and Levene’s test, respectively. When requirements for the parametric analysis were not met, the nonparametric Kruskal-Wallis test was performed. To view patterns in the filtered data matrix (i.e. data of samples and metabolite features), heatmaps with an agglomerative hierarchical clustering and Pearson correlation as distance metric were created from data in both the ESI+ and ESI- mode using the mixOmics library [72] in R.

## Results

### Metabolite identification

**Fig 2:**
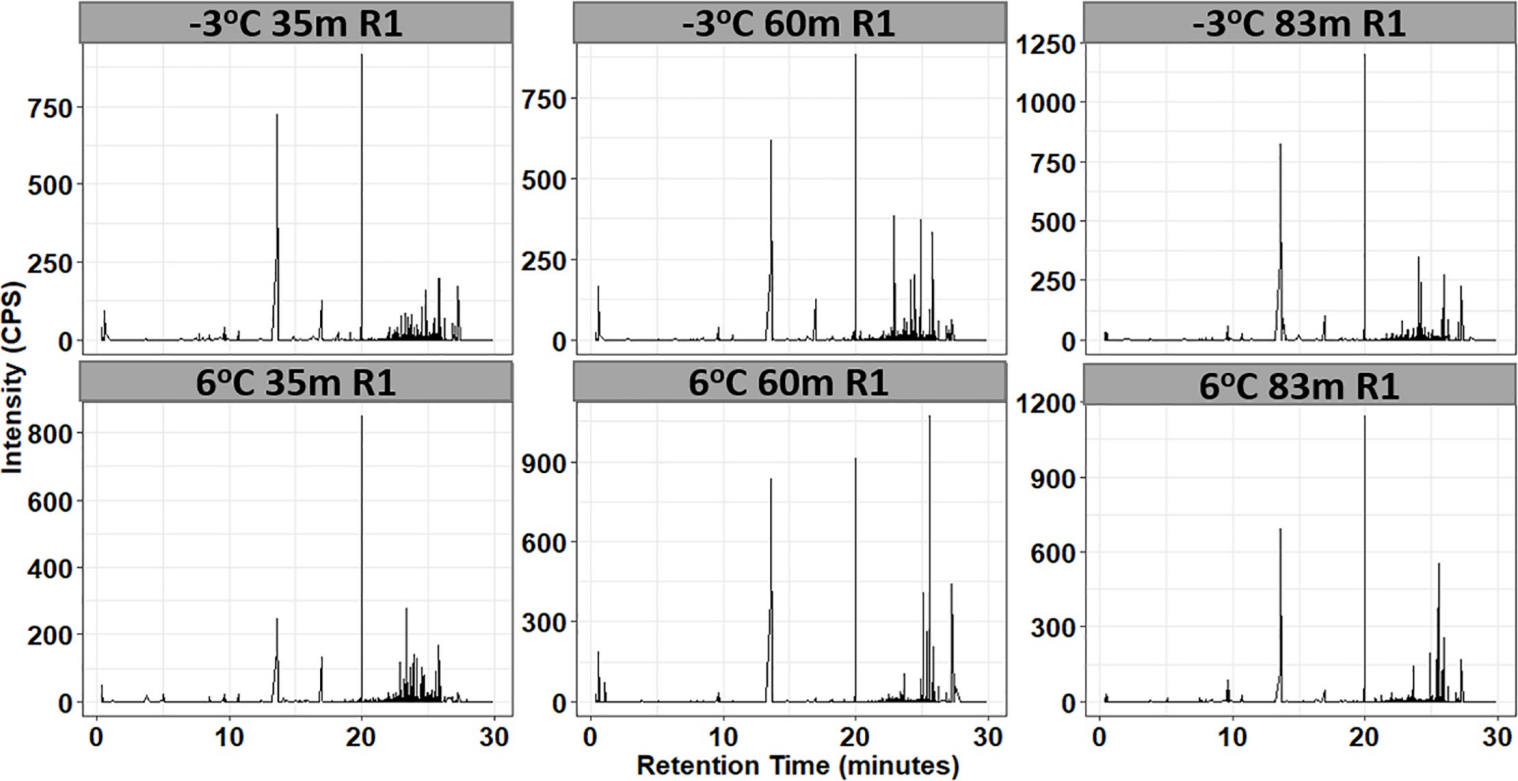
Representation UPLC chromatograms of metabolites from microbial cells in permafrost soil sample collected at the location 35m into the North Tunnel and incubated at -3°C and 6°C in ESI+ mode. R1 denotes the repetition 1 and intensity is measured in counts per second (CPS).

Data from both the ESI+ and ESI- modes were included because ionization efficiency (i.e. the amount of ions generated from a specific compound in the ionization source) can vary from compound to compound by several orders of magnitude [73]. A total of 10331 and 68736 metabolic hallmarks above the signal to noise ratio of 5 with unique retention time and m/z were detected in the ESI+ and ESI- mode, respectively. After filtering the dataset based on VIP scores (VIP>1.5), the metabolic traits were reduced to 27 and 54 in the ESI+ and ESI- mode, respectively, indicating that the metabolite products of the microbial cells were highly complex in chemical composition (see example in Fig 2). We however note that analysis was conducted on both the filtered and unfiltered datasets.

### Dimensionality reduction

**Fig 3:**
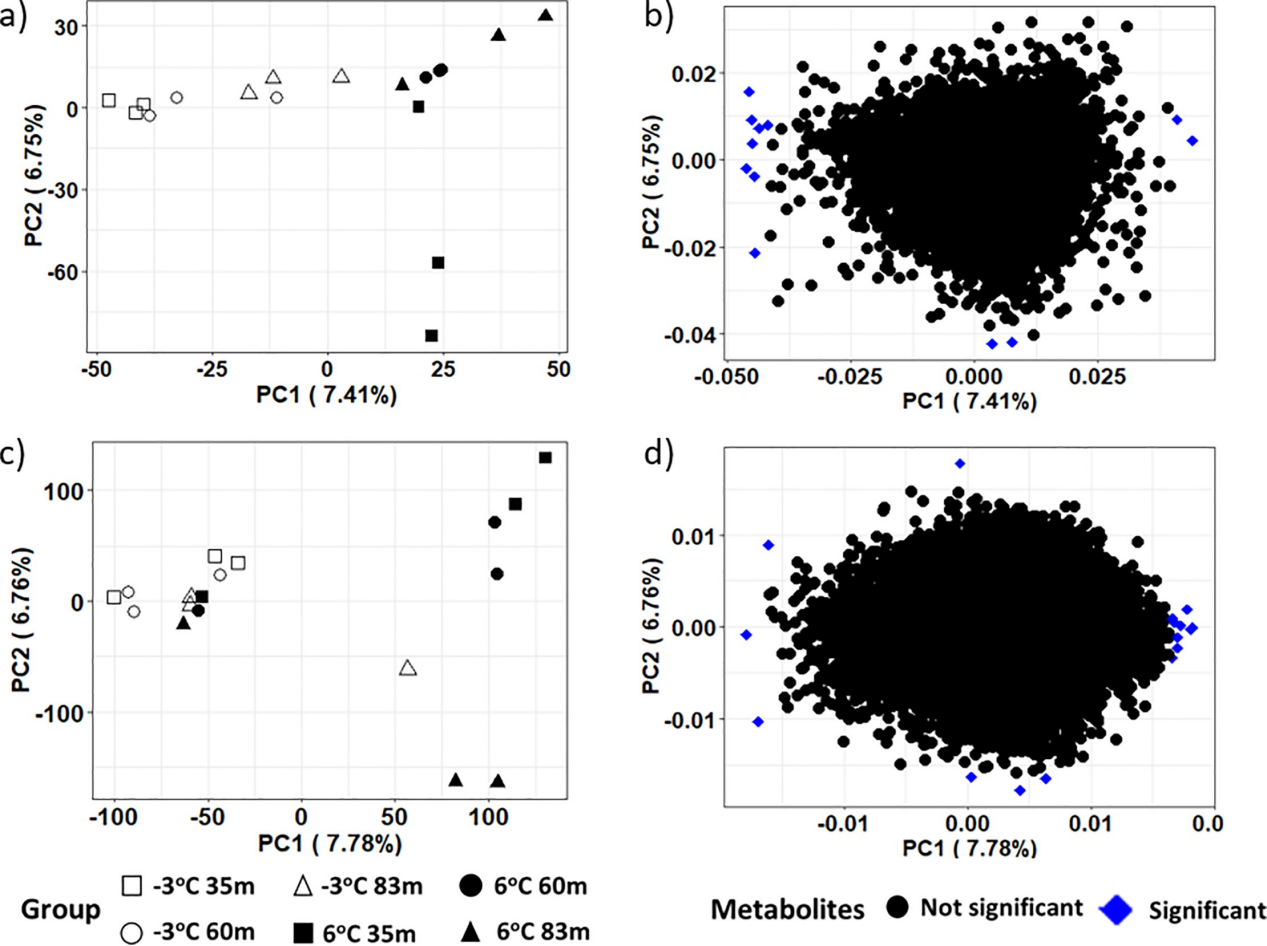
A 2-D plot of the first two principal component (PC) scores and loadings calculated from the unfiltered dataset of the metabolites in permafrost microbial cells at the various locations and thawed from -3°C to 6°C. Figures a and b are the PC scores and loading from the ESI+ mode and figures c and d are the PC scores and loadings from the ESI- mode respectively. Variables in the upper or lower 10% of the PCA loadings are the significant metabolites and these significant metabolites are in blue in the loading plots.

**Fig 4:**
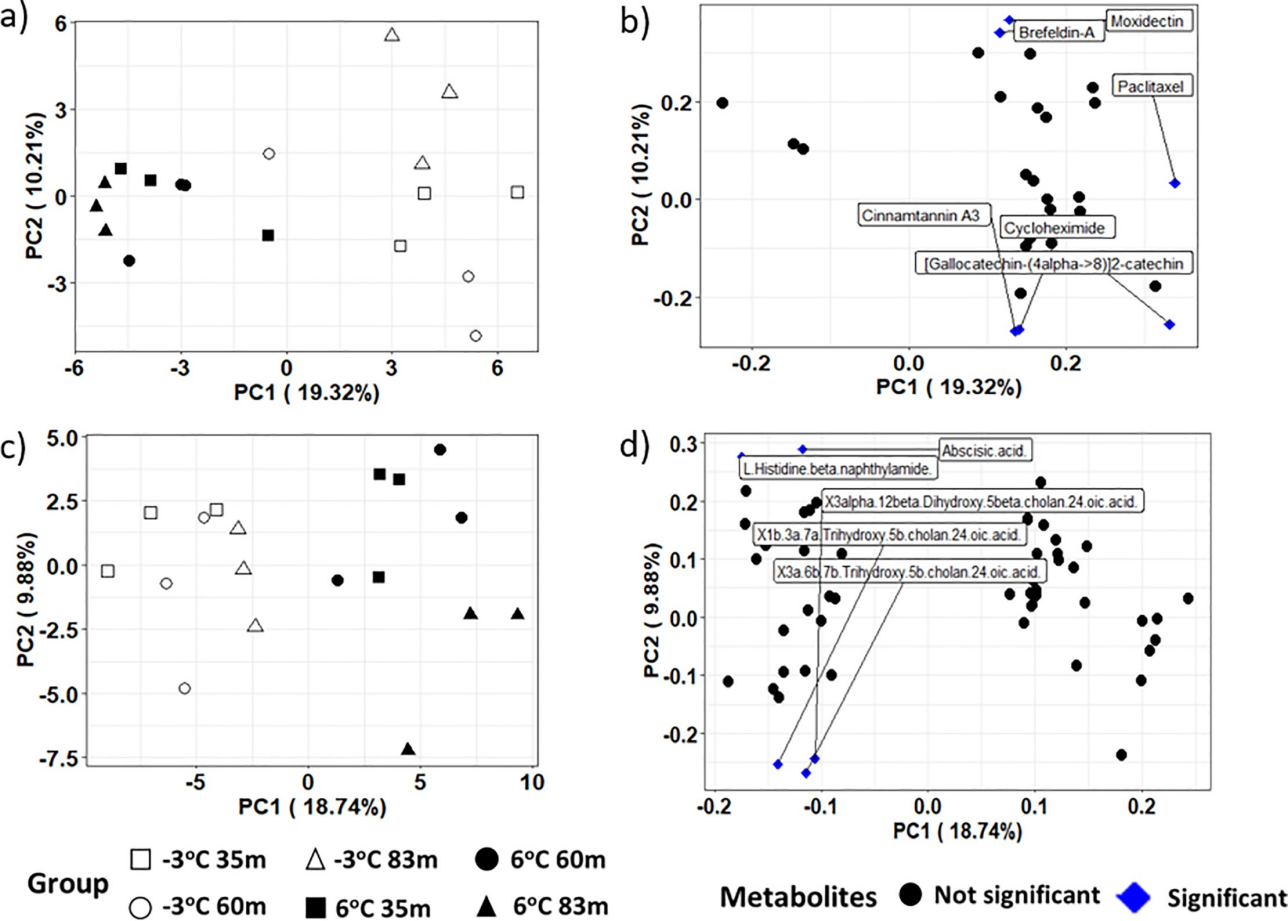
A 2-D plot of the first two principal component (PC) scores and loadings calculated from the filtered dataset of the metabolites in permafrost microbial cells at the three different sample locations and thawed from -3 ^o^C to 6 ^o^C. Figures a and b are the PC scores and loading from the ESI+ mode and figures c and d are the PC scores and loadings from the ESI- mode, respectively. Variables in the top or lower 10% of the PCA loadings are the significant metabolites and these significant metabolites are in brackets in the loading plots.

**Fig 5:**
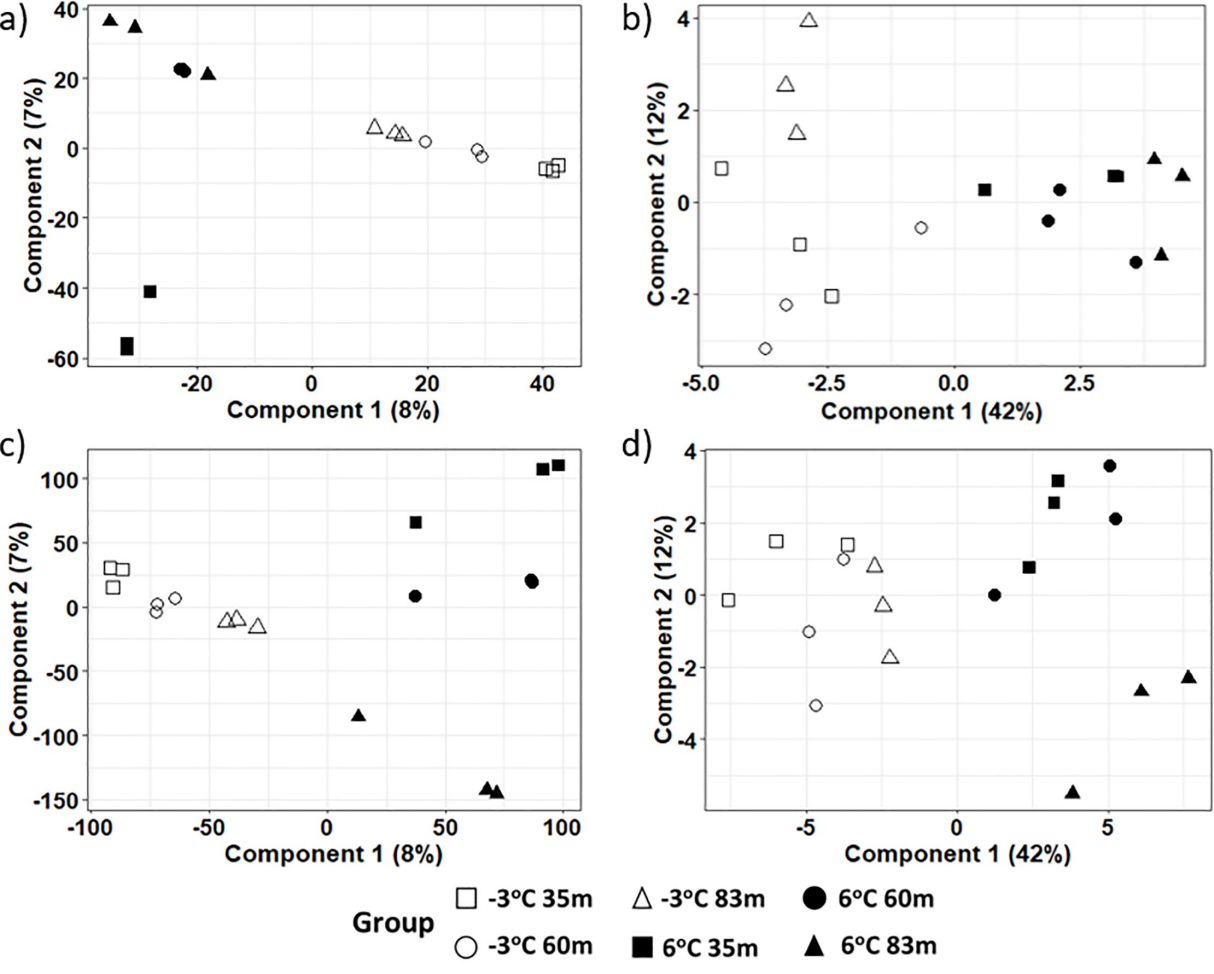
A 2-D plot of the first two components of the PLS-DA scores calculated from dataset of the metabolites in permafrost microbial cells at the various locations and thawed from -3 ^o^C to 6 ^o^C. Figures a and b are the PLS-DA scores from the unfiltered and filtered datasets, respectively, in the ESI+ mode and figures c and d are the PLS-DA scores from the unfiltered and filtered datasets, respectively, in the ESI- mode.

PCA results on the intensities of the metabolite products in both the ESI+ and ESI- modes revealed aggregation by thaw temperature and not location across the first principal component, indicating that temperature rather than location influenced the frozen and thawed metabolome (see Figs 3A, 4A and 4C for the ESI+ mode of the unfiltered dataset, ESI+ mode in the filtered dataset, and ESI- mode in the filtered dataset, respectively). A closer examination of the PCA based on location revealed some grouping of samples from the same location incubated at the same temperature. However, the pattern of grouping was not consistent in the ESI+ mode of the unfiltered dataset, ESI+ and ESI- modes in the filtered dataset (Figs 3A, 4A and 4C). The PCA result on the intensities of the metabolite products in the unfiltered ESI- mode (Fig 3C) did not show any particular differences in experimental groups. It is noteworthy that while PCA, as an unsupervised projection method, is useful at visualizing all information within a dataset, the results can sometimes be unsatisfactory due to the unsupervised nature of such analysis (see a detailed discussion of the PCA method’s advantages and disadvantages in Karamizadeh et al. [68]. To eliminate any non-specific effects of the operative technique, a Partial Least Square Discriminant Analysis (PLS-DA) was performed to sharpen the separation between groups. It is noted in Barker and Rayens [74] that PLS approaches are preferred over PCA when discrimination is the goal and dimension reduction is desired. Thus PLS-DA as a supervised approach was used in addition to the PCA methodology to determine similarities in metabolomics profiles between sample treatments. Regardless of ESI mode or whether the dataset was filtered or unfiltered, the PLS-DA illustrated a strong separation of samples by incubation temperature on the first component (Fig 5). The variation explained for the filtered datasets was much higher than for the unfiltered dataset (Fig 5B and 5D). When comparing samples within each incubation temperature, unfiltered metabolite profiles of permafrost incubating at -3°C clustered strongly by location when compared to the thawed permafrost (Fig 5A and 5C). Though some clustering by location was observed in the thawed samples, the variation was higher, perhaps because the microbial metabolism needed more time at 6°C to equilibrate (Fig 5A and 5C).

**Fig 6:**
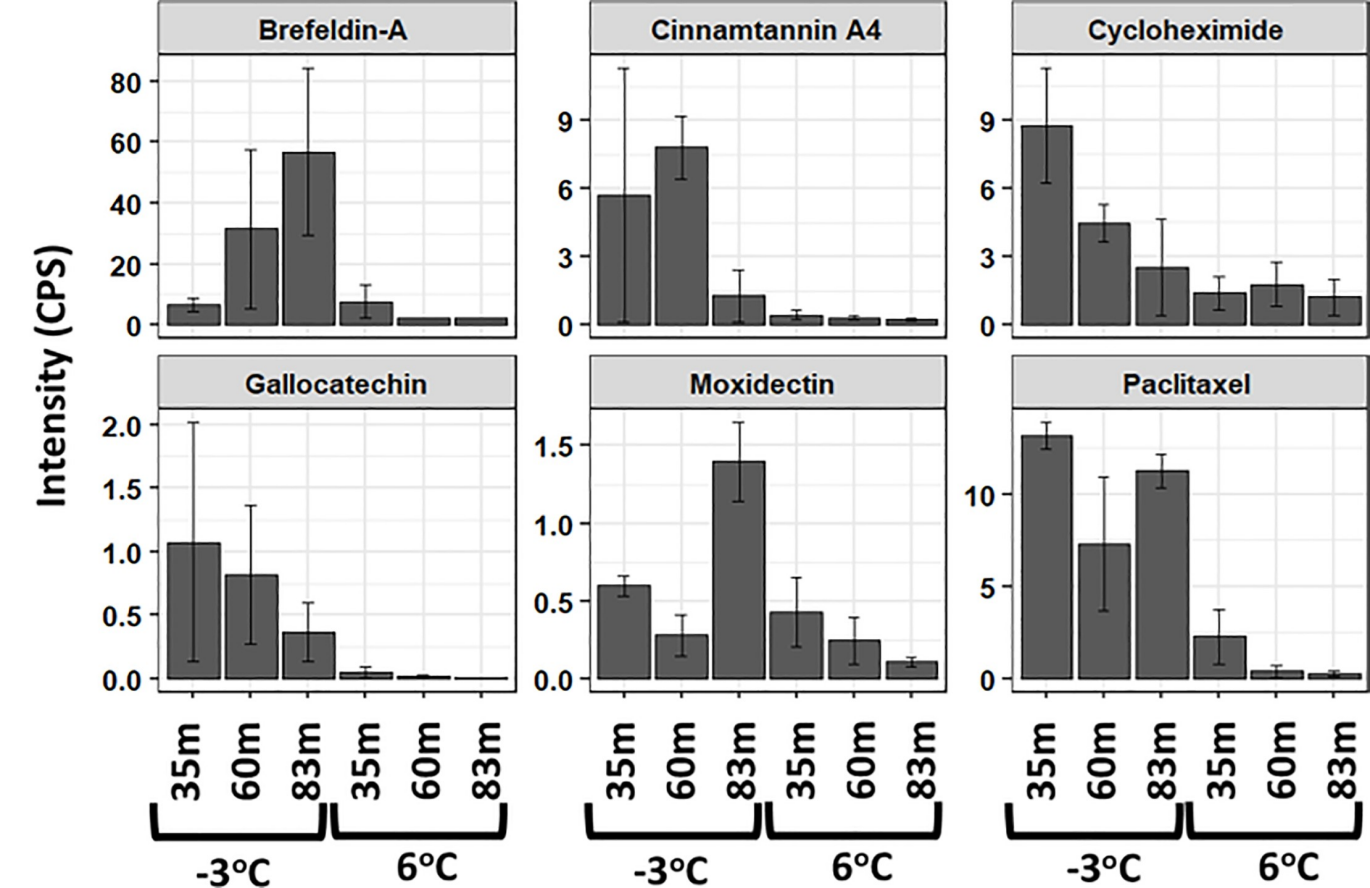
A bar chart of the significant metabolites driving discrimination groups in the PCA scores with their respective standard error over three replicates in the ESI+ mode. Important metabolites were identified as the top 10% of the PCA loadings in the filtered dataset.

**Fig 7:**
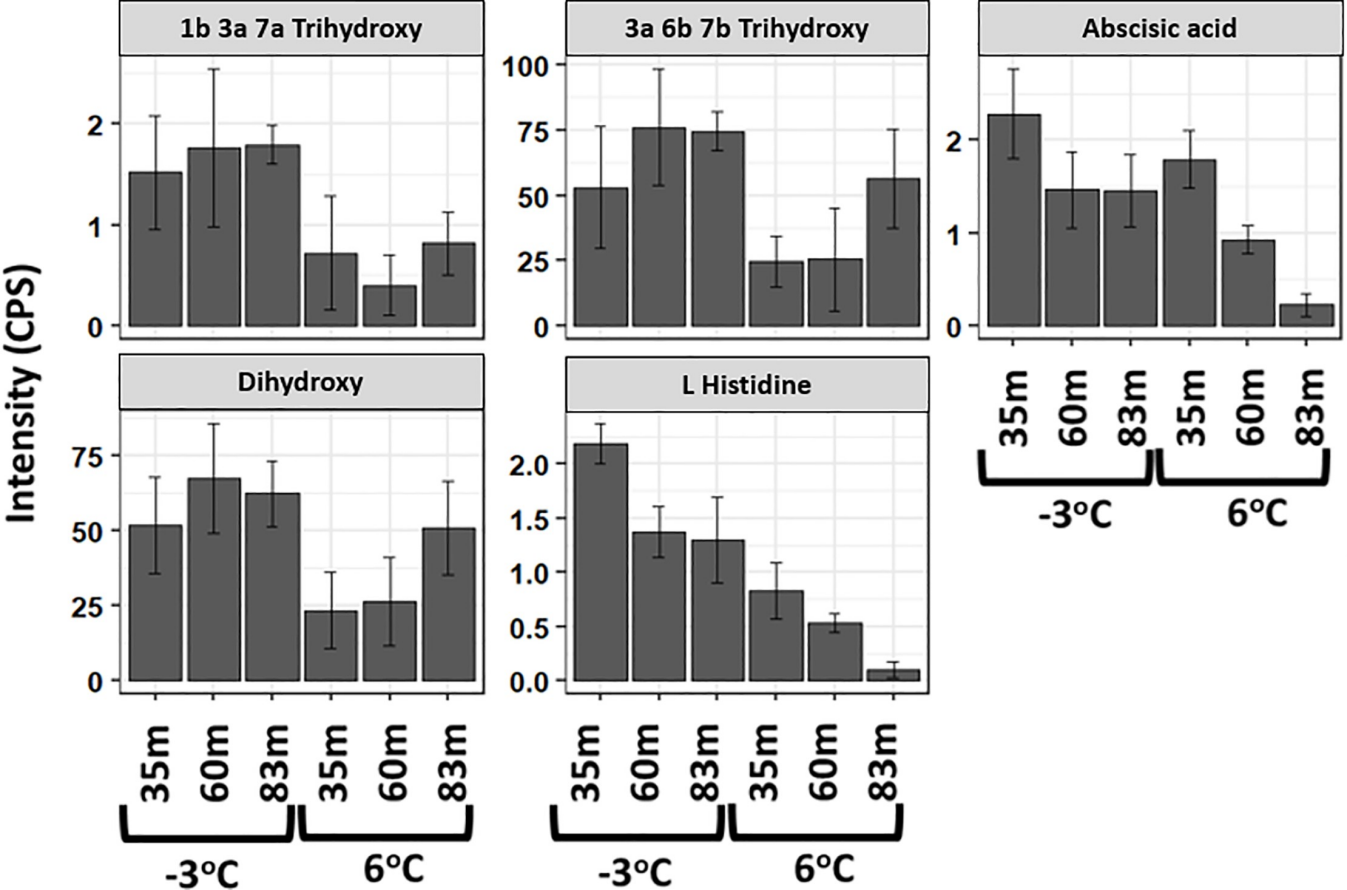
A bar chart of the important metabolites driving discrimination groups in the PCA scores with their respective standard error over three replicates in the ESI- mode. Important metabolites were identified as the top 10% of the PCA loadings in the filtered dataset. Metabolites “1b 3a 7a Trihydroxy”, “3a 6b 7b Trihydroxy”, and “Dihydroxy” were abbreviated for “1b 3a 7a Trihydroxy 5b cholan 24 oic acid”, “3a 6b 7b Trihydroxy 5b cholan 24 oic acid”, and “3alpha 12beta Dihydroxy 5beta cholan 24 oic acid”, respectively.

**Fig 8:**
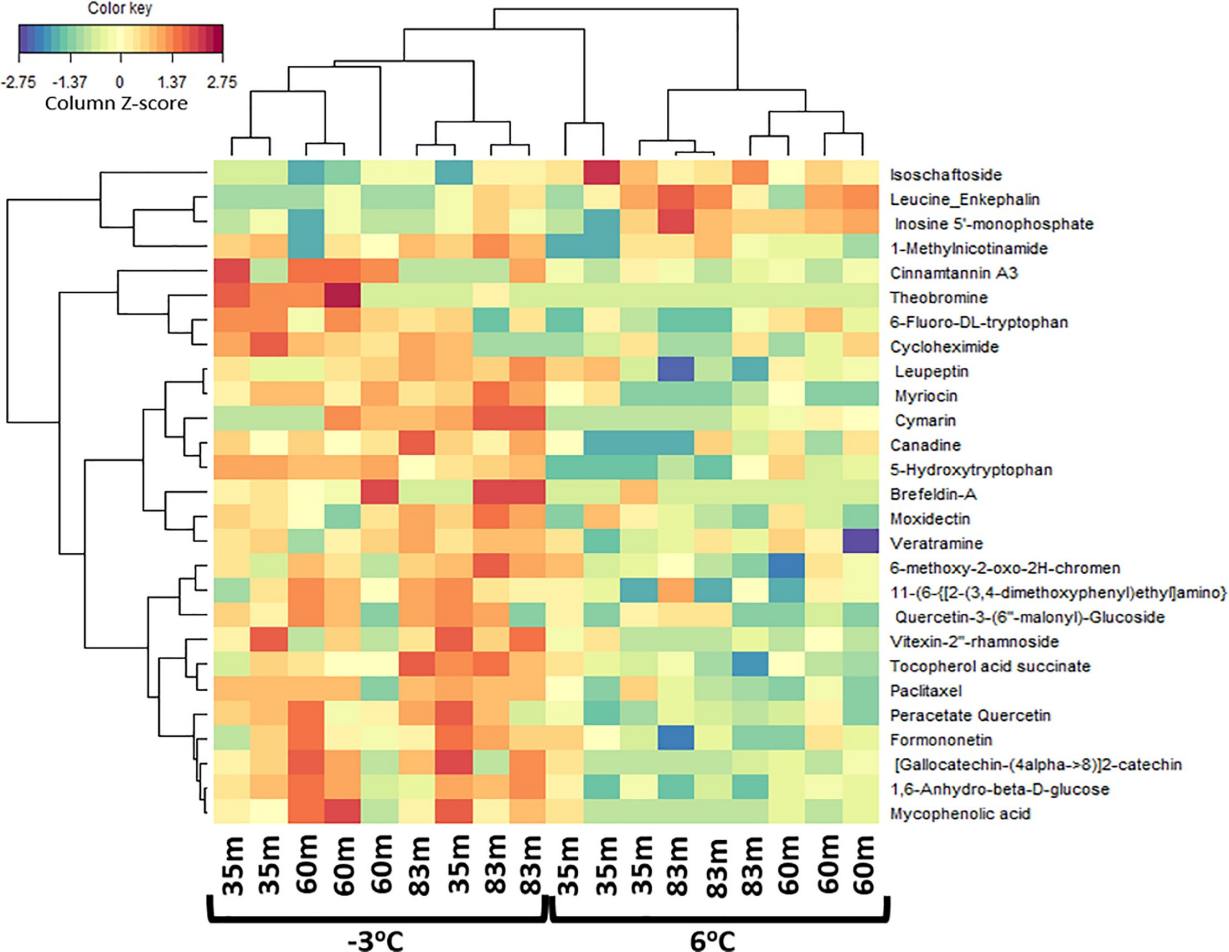
A heatmap illustrating from the hierarchical clustering analysis of metabolites in permafrost microbial cells at the three locations and thawed from -3 ^o^C to 6 ^o^C. The map shows a scaled Z-scores intensity values of 27 metabolites of the filtered dataset under the ESI+ mode. The negative (blue) value indicates an intensity that lies below the mean and the positive (red) values indicate an intensity that is above the mean. Metabolites “11-(6-{[2-(3,4-dimethoxyphenyl)ethyl]amino}” and “6-methoxy-2-oxo-2H-chromen” were abbreviated for “11-(6-{[2-(3,4-dimethoxyphenyl)ethyl]amino }-4-chloro-1,3,5-triazin-2-yl)-7” and “6-methoxy-2-oxo-2H-chromen-7-yl 2-O-acetyl-6-O-(6-deoxyhexopyranosyl) hexopyranoside” respectively.

**Fig 9:**
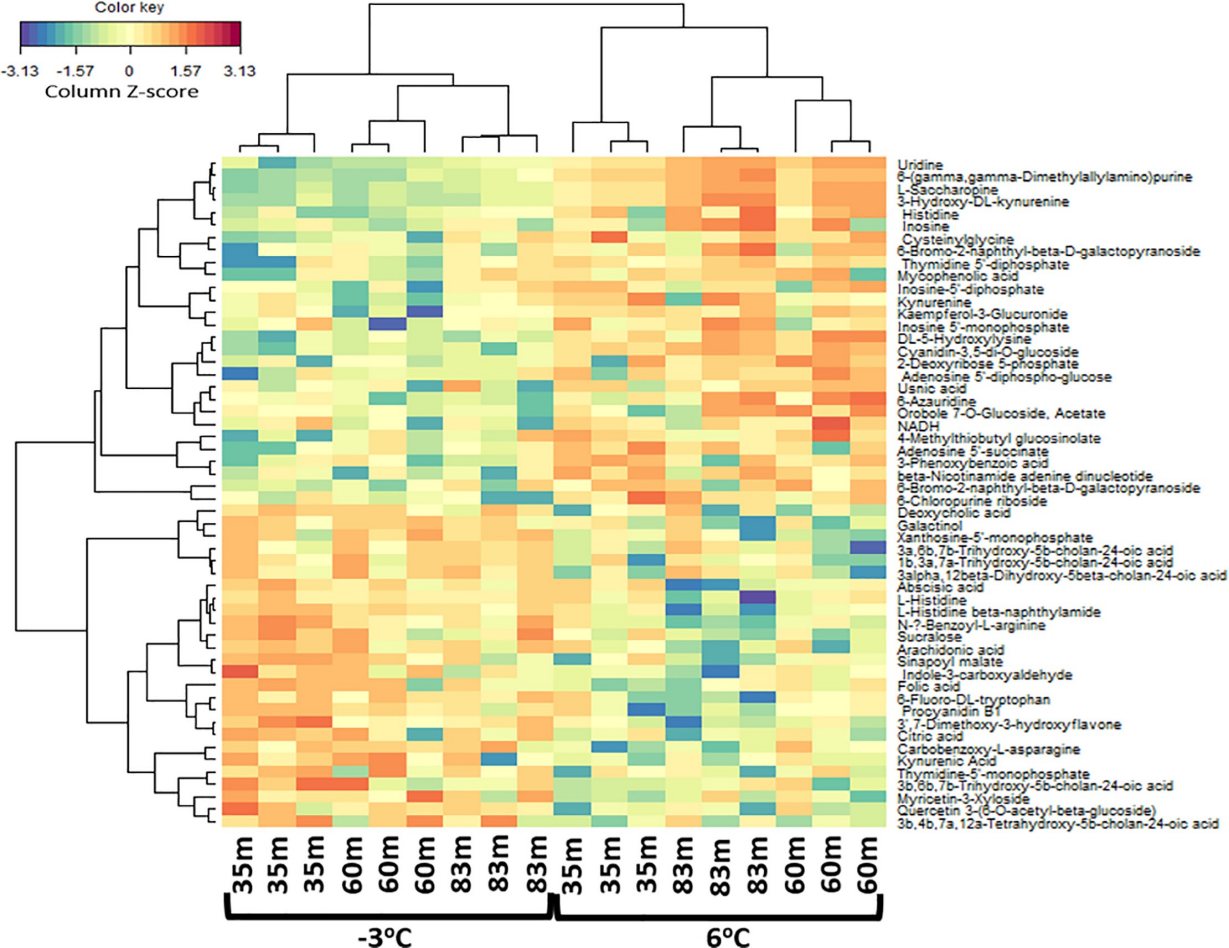
A heatmap illustrating from the hierarchical clustering analysis of metabolites in permafrost microbial cells at the three locations and thawed from -3 ^o^C to 6 ^o^C. The map shows a scaled Z-scores intensity values of 54 metabolites of the filtered dataset under the ESI- mode. The negative (blue) value indicates intensity that lie below the mean and the positive (red) values indicate an intensity that is above the mean.

Significant metabolites or variables with high PCA loadings (upper or lower 10%), which correspond to the variables that are most likely to discriminate groups in the PCA scores were identified. In the unfiltered dataset, we found 12 and 13 significant metabolites in the ESI+ and ESI- mode, respectively (Fig 3B and 3D). In the filtered dataset, 6 and 5 significant metabolites were found in the ESI+ and ESI- mode, respectively (Fig 4B and 4D). Additional analyses were only conducted on the significant metabolites in the filtered dataset because they reflect known metabolites found in online databases. For both ion modes, there was a decline in the significant metabolites’ intensity as thaw temperature increased (Figs 6 and 7). The only exception to the decline in metabolite intensity from -3°C to 6°C were Brefeldin-A and Moxidectin found in the 35 m and 65 m locations, respectively where the metabolite intensity remained approximately constant (Fig 6). In addition, heatmaps with hierarchical clustering based on individual sample and metabolic traits (27 in the ESI+ and 54 in the ESI- mode) in the filtered dataset revealed clustering by thaw temperature (Figs 8 and 9). Interestingly, in the ESI+ mode, the intensities of the metabolites in the frozen state were higher when compared to the thaw state, suggesting that microbial metabolism relating to known metabolites at sub-zero temperatures was more active (Fig 8). In the ESI- mode, both the frozen and thawed state induced increases in metabolite intensities, but for different processes (Fig 9). No single location appeared to have an increase of particular metabolites in either mode, except for the metabolites from the ESI+ mode in the 83 m samples incubating at -3°C (Figs 8 and 9).

### Statistical comparison of known metabolites

To understand the shift in metabolic changes due to the thaw temperatures and at our three different sample locations, various statistical approaches were utilized on the filtered and unfiltered datasets. Results from the statistical analyses (Table 1) and clustering illustration shown in the heatmaps (Figs 8 and 9) are consistent with our results from the PCA and PLS-DA analyses. These results provide a robust view of the biochemical structure in permafrost in response to a changing temperature and at different locations by pointing out that temperature is the main variable driving change in metabolic intensity. Independent of location, there was a statistically significant decline of metabolite intensity from the frozen to the thawed state (i.e. comparison of metabolite intensities between -3°C and 6°C) at α = 0.05 for all significant metabolites at ESI- mode (p-value equal to 0.027, 0.039, 0.004, 0.027, 0.02 for Abscisic acid, Dihydroxy, L histidine, 1b 3a 7a Trihydroxy, and 3a 6b 7b Trihydroxy, respectively) and half of the significant metabolites at ESI+ mode (Table 1) using paired samples Wilcoxon t-test (p = 0.035 for Cycloheximide, p = 0.042 for Gallocatechin, and p = 0.014 for Paclitaxel). With the exception of Moxidectin (F_2,6_ = 11.6, p = 0.009) and Abscisic acid (F_2,6_ = 14, p = 0.006), ANOVA results revealed that a statistically significant difference at α = 0.05 was not observed among locations for the intensity of the majority of the significant metabolites at the -3°C and 6°C incubation temperatures (Table 1). Only Moxidectin (p = 0.009) in the ESI+ mode and Abscisic acid (p = 0.006) in the ESI- mode were statistically significant at -3 ^o^C and 6 ^o^C respectively from the ANOVA results.

**Table 1:** P-Values for paired samples Wilcoxon t-test comparing significant metabolites at different temperatures (-3°C vs. 6°C) and ANOVA comparing the significant metabolites at 35m, 60m, and 83m at both temperatures respectively.

	Average Intensities						t-test p-values	ANOVA p-values (35, 60, and 83m)	
Significant Metabolites	-3°C 35m	-3°C 60m	-3°C 83m	6°C 35m	6°C 60m	6°C 83m	(-3°C vs. 6°C)	-3°C	6°C
**ESI+**									
Brefeldin-A	6.609	31.55	56.87	7.672	2.195	2.195	0.108	0.337	0.368^χ2^
Cinnamtannin A4	5.725	7.828	1.279	0.434	0.317	0.248	0.107	0.432	0.660
Cycloheximide	8.740	4.479	2.544	1.396	1.785	1.225	0.035*	0.151	0.687^χ2^
Gallocatechin	1.076	0.819	0.369	0.052	0.022	0.011	0.042*	0.745	0.619^χ2^
Moxidectin	0.599	0.282	1.398	0.428	0.246	0.109	0.098	0.009**	0.414
Paclitaxel	13.19	7.317	11.25	2.286	0.401	0.299	0.014*	0.236	0.278
**ESI-**									
Abscisic acid	2.285	1.470	1.457	1.792	0.926	0.226	0.027*	0.359	0.006**
Dihydroxy	51.90	67.44	62.41	23.38	26.37	50.81	0.039*	0.776	0.394
L Histidine	2.185	1.370	1.296	0.825	0.531	0.103	0.004**	0.126	0.054
1b 3a 7a Trihydroxy	1.520	1.764	1.793	0.723	0.404	0.818	0.027*	0.933	0.762
3a 6b 7b Trihydroxy	53.04	76.09	74.52	24.72	25.62	56.39	0.020*	0.657	0.376

* denotes significance at 0.05.

** indicates significance at 0.01.

χ^2^ implies p-Values for Kruskal-Wallis rank sum test when parametric assumptions are not met.

The significant metabolites are identified based on 10% of the maximum and minimum of the PCA loadings. Metabolites “1b 3a 7a Trihydroxy”, “3a 6b 7b Trihydroxy”, and “Dihydroxy” were abbreviated for “1b 3a 7a Trihydroxy 5b cholan 24 oic acid”, “3a 6b 7b Trihydroxy 5b cholan 24 oic acid”, and “3alpha 12beta Dihydroxy 5beta cholan 24 oic acid” respectively.

## Discussion

As mean air temperatures rise in high-latitude regions, permafrost thaw is projected to accelerate, dramatically altering the landscape [75]. It is unclear how biogeochemical processes will change during permafrost thaw. Metabolomics analysis provides critical information to determine the fate of biochemical processes and in turn permafrost functionality as thaw is induced from rising temperatures. In our study, the functional response of the permafrost microbiome to temperatures that simulated thaw depended more on the end-state thaw temperature rather than the location (i.e. age and depositional climatic and biogeochemical conditions). Specifically, two different analyses of filtered and unfiltered datasets aligned in that biochemical activity (as determined by two ion modes) in the permafrost was not significantly different between sites but rather by temperature. Metabolites from the thawed permafrost microbiome were similar regardless of their age and therefore the climatic and biogeochemical soil conditions during which the permafrost aggraded syngenetically.

The significant decline in known metabolites following data filtration was expected because current databases rely heavily on clinical strains of microorganisms. Though the filtered data represented only a portion of the dataset, they provided useful insight into known emergent functions. Interestingly, the majority of the significant metabolites identified in the PCA loadings synthesized by either a fungus or bacterium had antagonistic properties. For instance, Cycloheximide and Brefeldin-A are biocidal and antiviral compounds produced by the bacterium *Streptomyces noursei* and fungus *Penicillium brefeldianum* respectively [76, 77]. The presence of these metabolites at higher intensities in the frozen state suggest that the microorganisms living at sub-zero temperature employed antagonistic strategies for survival. Possible explanations of their decline following thaw include that the microorganisms making those antagonistic metabolites were outcompeted or that they no longer had an ecological need to survive by producing them. Moreover, some of the significant metabolites found in our frozen intact permafrost that have been found to be naturally produced by plants (e.g. [Gallocatechin-(4alpha->8)]2-catechin and Cinnamtannin A3) also have antibacterial properties [78–80], indicating a stressor on the growth of certain bacteria in the permafrost. We highlight the fact that certain metabolites are naturally produced by microbial fermentation of plant residues (see epicatechin production by the fermentation of green tea in Jiang et al. [81] which explains the presence of plant producing metabolites in our samples. Additionally, several bile acids (e.g. 3a 6b 7b Trihydroxy 5b cholan 24 oic acid, 1b 3a 7a Trihydroxy 5b cholan 24 oic acid, and 3alpha 12beta Dihydroxy 5beta cholan 24 oic acid) associated with bacterial overgrowth and inflammation in mammals [82] exhibited a significant decline in intensity as thaw temperature increased. The presence of these molecules suggests novel survival strategies for microorganisms present in intact permafrost. Using alternate ‘omics approaches, Hultman et al. [25] also found novel microbial strategies in intact permafrost collected from the Alaska Peatland Experiment near Fairbanks, Alaska, USA, including cold shock proteins and motility. Though Hultman et al. [25] did not detect antimicrobial compounds, the results from both studies support the notion that microorganisms sustained in frozen quiescence focus on survival rather than growth. Furthermore, as the thawed soil community shifts to a new form of metabolism, the decline in antagonistic metabolites may result in increased taxonomic diversity and possibly function because there is less competition among the members within the soil community. The changing function of the microbial community has important implications for the ecosystem relating to nutrient cycling, microbial survival strategies, and even pathogenicity.

Supporting evidence of growth induced during thaw includes the presence of several metabolites (e.g. Inosine and Nicotinamide adenine dinucleotides) which are critical for many cellular functions and the biosynthesis of major cell components [83, 84]. These metabolites displayed an increasing pattern in intensity following thaw, suggesting that the soil community was in a state poised to produce more cells and cellular components for growth and reproduction. Together, the decline in intensity of the metabolites with anti-microbial (or fungicidal) properties and the increase in intensity of critical compounds for cell biosynthesis serve as supporting evidence of an ecological shift from competition to mutualism resulting in an opportunity for cellular growth following thaw. Even in tundra systems [85, 86], warmer conditions are typically more favorable for most microorganisms, particularly those who were in a reduced metabolic state during a prolonged period of sub-zero temperature.

Ecological shifts could impact landscape level ecosystem processes such as organic matter decomposition and greenhouse gas production [87–89]. It remains uncertain what effect different interactive behaviors (i.e. mutualism, competition, predation, etc.) between soil microorganisms could have on the ecosystem. Wall and Moore [87] state that understanding these interactions will contribute to future management of ecosystems under scenarios of increasing human-derived physical, chemical, and biotic disturbances. As permafrost in the Arctic and Antarctic regions continue to thaw, it will become imperative to elucidate the interlinkage between organisms in order to accurately predict soil organism behaviors for ecosystem functioning. Our study not only provides critical knowledge on the fate of permafrost in response to the climate warming but also identifies gaps to attain the later objective.

The spatial variability of permafrost dynamics adds another layer of complexity on understanding and predicting environmental consequences of permafrost thaw [90]. Due to the intricacies that arise in including spatial dimensions in predictive models, there are relatively limited quantitative works on predicting organisms’ behaviors in permafrost. Our study alleviates this complexity by presenting the important variable for model construction and call for more collaborative studies between mathematical and biological disciplines.

## Conclusion

As subarctic and Arctic permafrost thaws at unprecedented rates, understanding the trajectory of microbial functions is critical to improve predictions of ecosystem services. Our findings are the first to reveal that the permafrost metabolome shifts similarly during thaw despite different temporal origins. This fluctuation of metabolic intensity, induced by microbial activity, could have a range of implications on important ecosystem processes (e.g. changes in plant functional traits, emergence of pathogens, degradation, etc.) as the climate continues to change. Our results illustrate how the soil microbial functionality may change from competition and antagonism in the frozen state to the production of cellular components and growth, hence contributing to major changes in ecosystem services in a projected warmer future Arctic. Metabolomics can serve as a powerful tool to understand altered metabolisms in changing permafrost systems.
